# A Flexible Bi-Stable Composite Antenna with Reconfigurable Performance and Light-Responsive Behavior

**DOI:** 10.3390/polym15061585

**Published:** 2023-03-22

**Authors:** Yaoli Huang, Cong Zheng, Jinhua Jiang, Huiqi Shao, Nanliang Chen

**Affiliations:** 1 Engineering Research Center of Technical Textiles, Ministry of Education, College of Textiles, Donghua University, Shanghai 201620, China; 2 Engineering Research Center of Digitalized Textile and Fashion Technology, Ministry of Education, Shanghai 201620, China; 3 Innovation Center for Textile Science and Technology, Donghua University, Shanghai 200051, China

**Keywords:** reconfigurable antenna, bi-stable substrate, radiation pattern, carbon nanotube film (CNTF), conductive silver paste (CSP)

## Abstract

An integrated solution providing a bi-stable antenna with reconfigurable performance and light-responsive behavior is presented in this paper for the first time. The proposed antenna includes a radiation layer with conductivity, which is integrated onto the bi-stable substrate. First, the effect of the radiation layer material and substrate layer parameters on antenna performance was studied. The experiment showed that an antenna with CNTF has a wider impedance bandwidth than one with CSP, namely 10.37% versus 3.29%, respectively. The resonance frequency increases gradually with the increase in fiber laying density and fiber linear density. Second, the influence of state change of the substrate layer on the antenna radiation pattern was studied. The measured results showed that the maximum radiation angle and gain of states I and II are at 90°, 1.21 dB and 225°, 1.53 dB, respectively. The gain non-circularities of the antenna at states I and II are 4.48 dB and 8.35 dB, respectively, which shows that the antenna has good omnidirectional radiation performance in state I. The display of the array antenna, which shows that the array antenna has good omnidirectional radiation performance in state A, with gain non-circularities of 4.20 dB, proves the feasibility of this bi-stable substrate in reconfigurable antennas. Finally, the antenna deforms from state I to state II when the illumination stimulus reaches 22 s, showing good light-responsive behavior. Moreover, the bi-stable composite antenna has the characteristics of small size, light weight, high flexibility, and excellent integration.

## 1. Introduction

Recently, the proposal and research of flexible, multifunctional antennas have greatly expanded the application of integrated antennas in flexible wearable devices such as sensors and actuators [1,2,3]. At the same time, the development of fifth-generation (5G) wireless necessitates the development of a flexible antenna with integrated structure and function. The concept of a reconfigurable antenna is a perfect demonstration of structural and functional integration [4,5]. As mentioned in the previous literature, the reconfigurable performance of an antenna is mainly reflected by the change in its radiation pattern [6]. At present, the reconfigurable performance of antennas has been realized by researchers through certain technical means. For example, Alfred et al. [7,8] prepared a reconfigurable antenna that can achieve reconfigurable changes in the antenna pattern through MEMS switching in its operating frequency. However, these antennas need an additional MEMS switch to control the antenna in addition to the antenna device itself, which greatly increases the weight of the antenna and limits its application. At the same time, these methods require continuous electrical stimulation to maintain the deformation. After removing the stimulation, the antenna structure immediately changes back to its initial state, which causes energy waste. Therefore, it is necessary to develop a composite antenna with reconfigurable structure and antenna function.

The bi-stable structure is used to solve the energy-waste problem, by achieving reconfigurable antenna performance. “Bi-stable” structure refers to two stable configurations that can be maintained in one of the stable states without the maintenance of external energy. A short stimulus can make it jump from one stable state to another stable state, and removing the stimulus leaves the steady state shape unchanged [9]. Researchers have prepared a flexible film with bi-stable characteristics, owing to its structural design as well as silicone oil dissolved in organic solvents [10,11,12]. However, this bi-stable structure has low stiffness, and the dissolution process requires a large number of organic solvents, which is not environmentally friendly and not suitable for mass production. At present, the external actuating forces for the response of intelligent structures are mainly smart-actuating [13,14,15,16,17] and mechanical-force-actuating [18,19]. Among them, light is a common, natural source of light and clean energy, which can be turned on and off at long distances [20,21]. This simple actuating method can play a great role in the shape transformation of bi-stable structures.

According to the existing reports, it is known that the bending angle of antennas has a significant impact on their working frequency and pattern [22]. Hu et al. [23] prepared the reconfigurable antenna by layering, which laid the foundation for the feasibility of the research in this paper. The results showed that the antenna has omnidirectional radiation when it is curled, with a gain of −1.52 dB, and directional radiation when it is unfolded, with a gain of 9.77 dB. However, these antennas require shape memory alloys and electricity as external actuating sources during the state-conversion process, and the antennas are too stiff to be widely used in smart wearable devices. In addition, researchers prepared a bi-layer encapsulated PCL-TPU shape memory composite structure by 4D printing [24]. The successful implementation of this technology will help people to expand the structural diversity of shape memory composites. Perhaps in the future, bi-stable structures with intelligent responses could be prepared directly by 4D printing [25]. Zheng Zhang et al. [26] prepared a bi-stable laminate using commercial silicone rubber-reinforced carbon fiber ply, and the state transition was made via gas actuation. However, the gas drive generally requires substantial air pressure to change its state. Therefore, to meet the needs of space detection and smart wearable devices, it is crucial and practical to design a light-driven, flexible, reconfigurable antenna with a simple fabrication method, deformable structure, low price, low energy consumption, and integrated structure and function. It has been reported that polydimethylsiloxane (PDMS) can be used as a good antenna substrate layer material due to its excellent optical properties and good hydrophobic and high dielectric constants [27,28,29]. Similarly, as reported in the References, polyimide (PI) fiber is widely used due to its high strength, low coefficient of thermal expansion (CTE), and high rigidity [30,31].

In this study, a bi-stable composite antenna is composed of a substrate layer and a radiation layer. First, the substrate layer, containing a functional layer and middle layer, is fabricated by antisymmetric laying and a high-temperature curing method. Next, the radiation layer is prepared with conductive material using screen-printing or laser-cutting technologies. Next, the antenna performance is studied by experimental methods. The reflection coefficient (S11) and the radiation pattern in state I and state II are tested. Lastly, the effects of the radiation layer material and substrate layer parameters on the antenna reflection coefficient are compared and analyzed.

## 2. Experimental Section

### 2.1. Materials

Carbon nanotube film (CNTF) with a thickness of 10–20 μm was purchased from Zhongke Times Nano Co., Chengdu, China. Conductive silver paste (CSP) (DJ002) was purchased from Maintenance Co., HongKong, China. PDMS material was commercially purchased (Sylgard 184 silicone elastomer) Dow Corning., Midland, MI, USA. with a viscosity of 5500 mPA·s. PI fibers with different linear densities were purchased from Jiangsu Xiannuo New Material Technology Co., Ltd., China. PI electro-spun membranes with thicknesses of 80 μm were supplied by the Hunan Institute of Engineering.

### 2.2. Preparation of the Bi-Stable Substrate Layer

**Figure 1:**
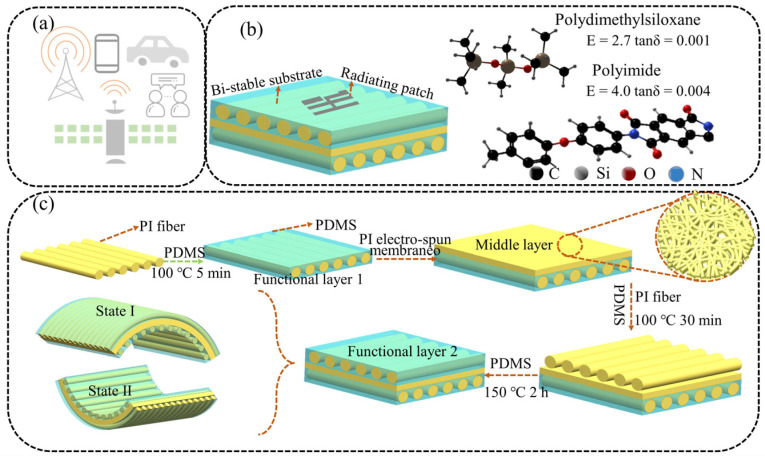
(**a**) Schematic of the potential applications of flexible film antennas. (**b**) Structural schematic of a micro-strip antenna. (**c**) Design and preparation of a reconfigurable substrate with bi-stable characteristics.

As shown in Figure 1a, flexible thin-film antennas have important applications in interpersonal communication, satellite communication, Bluetooth, etc. Figure 1b demonstrates a bi-stable composite antenna with light-responsive and reconfigurable performance, which provides new reference and theoretical data for the long-term application of reconfigurable flexible electronic devices. As shown in Figure 1b, a complete antenna is composed of a substrate layer and a radiation layer. In particular, the substrate layer has a direct influence on the reconfigurable performance of the antenna. Therefore, we first discuss the preparation method for the reconfigurable substrate with bi-stable characteristics.

Figure 1c shows a schematic for designing and fabricating a bi-stable substrate layer for the antenna. It can be seen that the bi-stable substrate layer consists of a middle layer and a functional layer. The functional layer includes Functional layer 1 and Functional layer 2, which are arranged in an anti-symmetric method. The bi-stable substrate layer is pre-cured using the residual thermal stress caused by the large CTE difference between the PI fiber and PDMS during high-temperature curing and the arrangement of antisymmetric layers.

It is worth noting that the PI electro-spun membrane should be pasted at the PDMS curing temperature of 100 °C and a curing time of 5 min. At this time, the PDMS is pre-cured, but it is not completely cross-linked. The PI electro-spun membrane can be pasted onto Functional layer 1 by the viscosity of PDMS, to prevent slipping. The bi-stable substrate layer will maintain a stable structure when the sample is taken out at room temperature (state I). It can reach another stable structure (state II) through external stimulation. Significantly, in previous studies, we have proved that the oriented-PI-fiber-reinforced PDMS film has bi-stable characteristics and demonstrates a close relationship between fiber laying densities and fiber linear densities [32].

### 2.3. Preparation of the Radiation Layer

**Figure 2:**
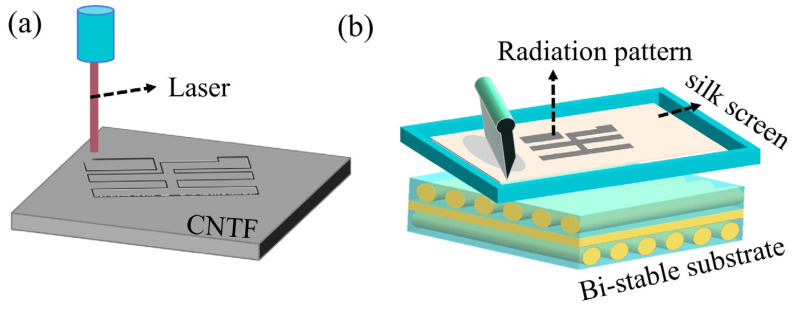
(**a**) Schematic diagram of laser cutting CNTF. (**b**) Schematic diagram of the screen-printing process.

The antenna radiation layer was prepared by screen printing and laser-cutting, as shown in Figure 2. First, the CNTF radiation layer was cut from the commercially available CNTF film with laser cutting technology and then pasted on the bi-stable substrate during the curing process. The process used a cutting power of 3 W and 5 cutting times.

Next, the CSP radiation layer was printed directly onto the cured bi-stable substrate using screen printing technology. At this time, 300 mesh screen cloth was used to penetrate the conductive silver paste.

### 2.4. Characterization

The morphological features of the radiation layer were investigated by a field-emission scanning electron microscope (FE-SEM 7500F, JEOL Technology and Trade Co., Ltd., Beijing, China). A UV light source with a wavelength of 395 nm was used to actuate the soft actuator, by means of a UV curing machine (SJUV3D-104, Shengju Machinery Automation Co., Ltd., Shanghai, China). A thermogravimetric analyzer (TGA8000, PerkinElmer Enterprise Management Co., Ltd., Shanghai, China) was used to test the high-temperature thermal stability of the radiation layer. Carbon nanotube films were cut by a portable micro-engraving machine (K6PRO, Shanghai Diaotu Industrial Co., Ltd., Shanghai, China).

The Tru EBox Workstation (01RC, LinkZill Corporation, Shanghai, China) was used to test the capacitance of the substrate layer. The reflection coefficient (S11) of all antennas was tested using a network analyzer (Keysight E5071C, Keysight Technology Co., Ltd., Shanghai, China) with open-short-load calibration. The radiation pattern made by the rotation angle position of the horn antenna and the testing antenna was tested using the network analyzer.

## 3. Results and Discussion

### 3.1. Effect of Radiation Layer Material on Antenna Performance

**Figure 3:**
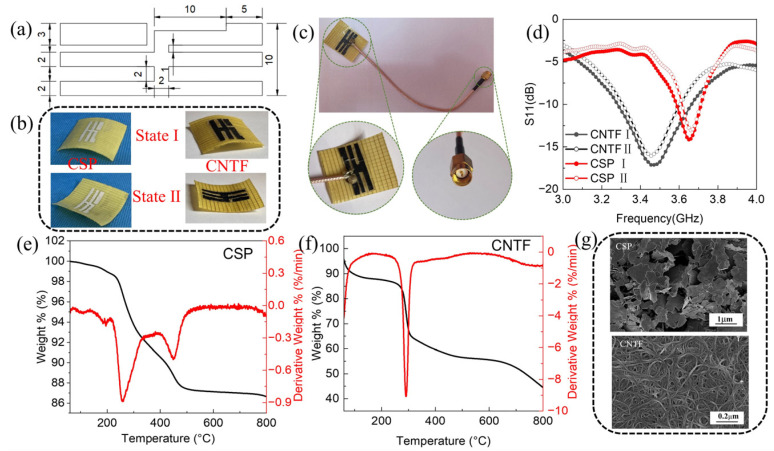
(**a**) Digital image of the geometry and size of as-fabricated reconfigurable antennas. (**b**) The physical images of the CSP-based and CNTF-based bi-stable antennas in state I and state II, respectively. (**c**) Physical diagram of the CNTF-based bi-stable antenna with a coaxial line feed. (**d**) Reflection coefficient (S11) of bi-stable antennas with different radiation layer materials in two states. (**e**) TG curves of conductive silver paste. (**f**) TG curves of carbon nanotube film. (**g**) SEM image of the radiation layer material.

The development of fifth-generation (5G) wireless will meet the basic requirements of small size, light weight, flexibility, and low price. At the same time, 2.1 GHz and 3.5 GHz are receiving more and more attention as the mainstream frequency bands for 5G network deployment. The pattern and conductivity of an antenna’s radiation layer play a decisive role in its electromagnetic performance. The radiation layer pattern and dimensions used in this paper are shown in Figure 3a. The bi-stable composite prepared in this section was cut to a size of 40 mm × 40 mm × 0.35 mm to obtain a reconfigurable substrate of the first state (state I) and the second state (state II).

Carbon nanotube film (CNTF) is a relatively mature conductive film material which, owing to the in-depth study and maturity of its production process, is becoming more and more popular [33,34]. In addition, conductive silver paste (CSP) is also very common in various electronic devices, due to its low price and mature formula [35]. To compare these, this paper investigates and analyzes the effect of using CNTF and CSP as radiation layer materials on antenna performance. Figure 3b shows physical pictures of the CSP-based and CNTF-based bi-stable composite antennas in states I and II. Figure 3c shows the physical diagram of a CNTF-based bi-stable antenna with a coaxial line feed. One of the enlarged images is of the connection between the antenna radiation layer and the coaxial line and the other is of the SMA interface connected to the test end.

Figure 3d shows the reflection coefficients of bi-stable antennas with different radiation layer materials in two states. First, it can be seen that the S11 curves of the antennas with the same radiation layer material coincide in state I and state II. This indicates that the stable-state changes do not have a large impact on the reflection coefficient and resonant frequency. However, it can be seen from Figure 3d that the change in radiation layer material does affect the resonant frequency and reflection coefficient of the antennas. It can be seen that the resonant frequency and reflection coefficient of the bi-stable antenna with CNTF as the radiation layer material are 3.47 GHz and −17 dB, respectively. In comparison, the resonant frequency and reflection coefficient of the bi-stable antenna with CSP as the radiation layer material are 3.65 GHz and −15 dB, respectively. This is mainly because the CNTF radiation layer material has better conductivity. The resistivity of the CSP radiation layer is 5 × 10^−5^ Ω·cm, while the resistivity of the CNTF radiation layer is 5 × 10^−4^ Ω·cm.

At the same time, impedance matching efficiency and impedance bandwidths are also important indexes for evaluating antenna performance, and are thus worth discussing and analyzing. When the impedance matching efficiency is closer to 100%, the antenna is in an ideal state. Similarly, when the impedance matching efficiency is closer to 0%, the antenna impedance is completely mismatched.

As shown in Figure 3d, all antennas with different radiation layer materials at two states have a reflection coefficient of <−10 dB, indicating the impedance matching efficiency of all antennas is greater than 90%, which meets the application requirements of antennas. However, the impedance matching efficiency of the antenna with the CNTF radiation layer is closer to 100%, which is closer to the ideal state.

The measured characteristics indicate that the resonant frequency of the antenna with the CNTF radiation layer is f = 3.47 GHz, and the impedance bandwidths (S11 ≤ −10 dB) are 10.37% (3.29–3.65 GHz). The resonant frequency of the antenna with the CSP radiation layer is f = 3.65 GHz, and the impedance bandwidths (S11 ≤ −10 dB) are 3.29% (3.59–3.71 GHz). This indicates that the antenna with the CNTF radiation layer has wider impedance bandwidths than the one with CSP. In summary, the bi-stable antenna with the CNTF radiation layer has a smaller reflection coefficient, higher impedance matching efficiency, and higher impedance bandwidths. Meanwhile, the resonant frequency of the bi-stable antenna with the CNTF radiation layer is closer to 3.5 GHz, which is more in-line with the use range of a 5G network than that of the antenna with CSP. Since the radiation layer is a light-driven heat transfer layer, it will directly contact the heat generated by the light source during the subsequent light response stimulation process. The study of thermal stability is therefore very necessary. Figure 3e,f shows the TG curves of CSP and CNTF. It can be seen that the mass loss rates of CSP and CNTF are 1.71% and 12.98%, respectively, at 50–230 °C, which indicates that they have good thermal stability. In addition, Figure 3g shows the SEM morphology of the CSP and CNTF layers, from which the sheet structure of the CSP and the tubular structure of the CNTF can be observed clearly. This shows that the radiation materials themselves have no defects and will not produce errors in the experimental results, nor affect the testing performance.

### 3.2. Effect of Substrate Layer Parameters on Antenna Performance

**Figure 4:**
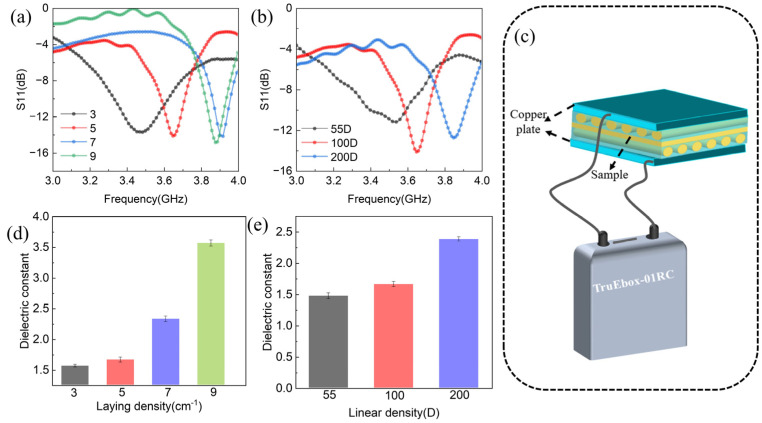
(**a**) Reflection coefficients (S11) of reconfigurable antennas with different fiber laying densities in state I. (**b**) Reflection coefficients (S11) of reconfigurable antennas with different fiber linear densities in state I. (**c**) Schematic diagram of the capacitance testing method. (**d**) Dielectric constants of substrate layers with different laying densities. (**e**) Dielectric constants of substrate layers with different linear densities.

As mentioned earlier, a complete antenna is composed of the substrate layer and radiation layer, so the impact of changes to the substrate layer parameters on the antenna performance is also well worth studying and exploring. To make a comparative test, this paper prepared a bi-stable substrate with different fiber laying densities and fiber linear densities. The radiation material used for all of the contrast antennas in this section was CSP. Fiber laying densities refer to the number of fibers per unit length of the functional layer. Linear density is a textile term that means the fineness of the fiber. Its unit is denier/D, which refers to the mass (in grams) of fibers with lengths of 9000 m. Figure 4a,b shows the reflection coefficient of bi-stable antennas with different fiber laying densities and fiber linear densities in state I. It can be seen that the resonance frequency of the antenna increases gradually with the increase in fiber laying density and fiber linear density. Conversely, the impedance bandwidth decreases gradually with the increase in fiber laying density and fiber linear density. We speculate that this is caused by the change in the dielectric constant of the substrate layer, because the substrate is generally required to be non-conductive in antenna design, and the dielectric constant is often used to characterize the degree of non-conductivity of the material. To verify this conjecture, we looked at the dielectric constant of the substrate layer with different fiber laying densities and fiber linear densities by inferring it from the capacitance values.

First, a schematic diagram of the substrate layer capacitance testing method is shown in Figure 4c. Second, because the substrate layer is not conductive, the following formula can be used to derive the dielectric constant of the substrate layer.

(1) $$\varepsilon_{r}=\frac{C\times d}{\varepsilon_{0}\times S}$$

where $\varepsilon_{0}$ is the vacuum dielectric constant, d is the thickness of the substrate layer, S is the contact area, $\varepsilon_{r}$ is the substrate layer dielectric constant, and C is the capacitance. The dielectric constants of the substrate layers with different fiber laying densities and fiber linear densities are shown in Figure 4d,e. It can be seen from here that the dielectric constant increased with the increase in fiber laying density and fiber linear density. Combined with Figure 4a,b, it can be found that the increase in the dielectric constant of the substrate layer drives the resonant frequency of the antenna to be larger. This phenomenon can be explained and verified by the following formula:

(2) $$f_{0}=\frac{c}{\lambda_{0}}\sqrt{\varepsilon_{r}}$$

where c is the speed of light, λ_0_ is the wavelength of free space, and $\varepsilon_{r}$ is the substrate layer dielectric constant. Therefore, the resonant frequency of the bi-stable antenna can be adjusted by the dielectric properties of the substrate layer in theoretical practice. However, it can be seen that the resonant frequency of the laying densities of 9 cm^−1^ and 7 cm^−1^ does not change much, which may be because the influence on the resonant frequency of the antenna is not obvious when the dielectric constant is too large. In addition, the change in laying density has little effect on the reflection coefficient of the antenna, but the fiber linear density seems to have a great influence on the reflection coefficient of the antenna. This is because the increase in fiber linear density increases the thickness of the bi-stable antenna’s substrate layer.

### 3.3. Reconfigurable Performance Analysis of Bi-Stable Antennas

**Figure 5:**
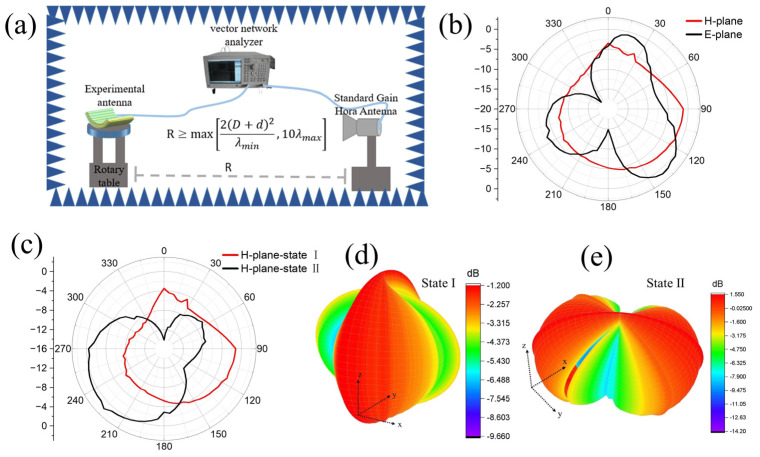
(**a**) Schematic diagram of the normalized radiation patterns. (**b**) Radiation patterns of the E- and H-planes of the bi-stable antenna at state I. (**c**) Radiation patterns of the H-plane of the bi-stable antenna at states I and II, respectively. (**d**) 3D radiation pattern of state I. (**e**) 3D radiation pattern of state II at 3.5 GHz.

The normalized radiation patterns and gain of an antenna are also important indexes to measure its electromagnetic performance, especially its reconfigurable performance. Therefore, it is very important to study the changes to the bi-stable antenna’s radiation pattern in different states. Figure 5a shows the testing scheme of the normalized radiation pattern. It can be seen that the whole testing system is composed of a network analyzer, standard horn antenna, rotary table, experimental antenna, and microwave anechoic chamber. The experimental antenna is placed on the rotary table to measure the gain values at different angles. The actual gain of the measured antenna is calculated from the standard gain antenna and its calibration gain, as shown below:

(3) $$G=G_{s}+(G_{x}-G_{w})$$

where G is the actual gain of the measured antenna, $G_{s}$ is the gain calibration value (7.5 dB) of the standard gain antenna at 3.5 GHz, $G_{x}$ is the receiving gain of the experimental antenna at 3.5 GHz, and $G_{w}$ is the receiving gain (−27.39 dB) of the calibrated standard gain antenna at 3.5 GHz. According to the relationship between the experimental antenna and the electromagnetic field direction of the test environment, the expression methods of E-plane and H-plane come into being. Among them, the E-plane is also called the electric plane, which refers to the direction plane parallel to the direction of the electric field. The H-plane, also known as the magnetic plane, refers to the direction plane parallel to the direction of the magnetic field. Figure 5b shows the E-plane and H-plane radiation patterns of state I at 3.5 GHz. It can be seen from Figure 5b that the radiation pattern of state I in the electric field plane (E-plane) is quasi-octagonal, and the radiation pattern in the magnetic field plane (H-plane) is closer to a circle, which obtains good omnidirectional radiation. The experimental results show that the maximum radiation angles of the E-plane and H-plane are at 135° and 90°, respectively. The measured gains of the antenna are 1.1 dB and 1.21 dB, respectively.

Figure 5c shows the H-plane radiation pattern of the bi-stable antenna in states I and II at 3.5 GHz. It can be found that the radiation pattern of the bi-stable antenna also changes accordingly with the change between state I and II. The experimental results show that the maximum radiation angles of states I and II are at 90° and 225°, respectively. The measured gains for states I and II are 1.21 dB and 1.53 dB, respectively. Gain non-circularity is often used to describe the omnidirectional radiation performance of the antenna. The following formula is a method for calculating gain non-circularity.

(4) $$G_{R}=max\left(\left|\left.G_{max}-G_{avg}\right|\right.,\left|\left.G_{min}-G_{avg}\right|\right.\right)$$

where $G_{R}$ represents the antenna gain non-circularity and $G_{max}$, $G_{min}$, and $G_{avg}$ represent the maximum gain, minimum gain, and average gain of the antenna in the H-plane.

The gain non-circularities of the antenna at states I and II are 4.48 dB and 8.35 dB, respectively. This shows that the bi-stable antenna has good omnidirectional radiation performance in state I. This is because the change to antenna structure causes a change in radiation direction and intensity of the radiation layer. We speculate that this is because the antenna radiation layer in state I is flat, while the antenna radiation layer in state II is bent, and the corresponding current radiation intensity is weakened in a bending direction. As a result, the main current transmission direction of the antenna is deflected; that is, the pattern of the same antenna is different under different structures, so it can be considered reconfigurable. At the same time, it can be seen from the 3D radiation pattern of Figure 5d,e that the gain of state I and II also changes due to the deformation of the antenna structure, but both have good gain performance.

**Figure 6:**
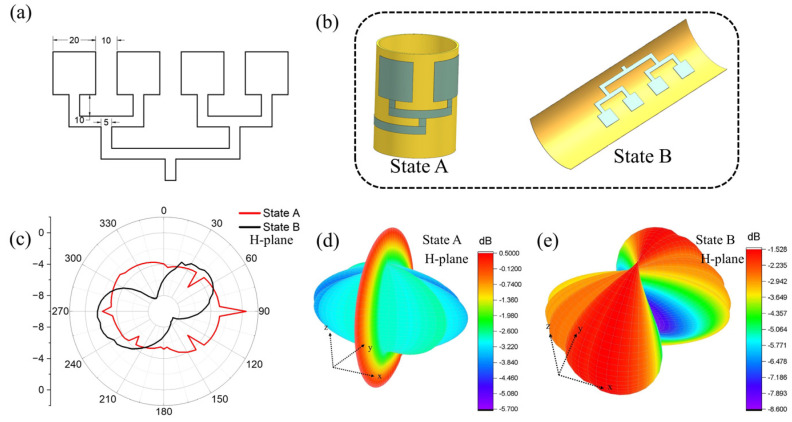
(**a**) Digital image of the geometry and size of the antenna array’s radiation layer. (**b**) Schematic diagram of the pattern of the bi-stable antenna array. (**c**) Radiation patterns of the H-plane of the bi-stable antenna array at states A and B. (**d**) 3D radiation pattern of state A. (**e**) 3D radiation pattern of state B.

To improve the transmission capacity of the antenna, reduce the cost of building a network in high-traffic areas, and meet the applications of various occasions, several single antennas with the same resonant frequency can together be used as the basic unit and, with reasonable planning, be combined in space to form an array antenna. The main function of the antenna array is to strengthen and improve the directivity of the radiation field and to strengthen the intensity of the radiation field. The bi-stable composite material prepared in this section was cut to a size of 150 mm × 80 mm × 0.35 mm to obtain a reconfigurable substrate of the first state (curled) and the second state (unfolded). Figure 6a shows the specific size and shape of the antenna radiation layer array.

As shown in Figure 6a, the distance between each single antenna element is 10 mm. Figure 6b shows the schematic diagram of the pattern of the reconfigurable antenna array. As shown in Figure 6b, the size of the radiation layer is just one cycle around the first stable-state substrate layer, which is defined as state A; the second stable-state is defined as state B. Figure 6c shows the radiation patterns of the H-plane of the reconfigurable antenna array at states A and B

It can be seen that in state A, the gain value of the reconfigurable array antenna in each direction does not change much, while in state B, the reconfigurable array antenna shows strong directional radiation characteristics in the 90° direction. The gain non-circularities of the array antenna at states A and B are 3.47 dB and 4.20 dB, respectively. This shows that the array antenna has good omnidirectional radiation performance in state A. This further proves the possibility for reasonable application of bi-stable substrates in the reconfigurable performance of array antennas.

### 3.4. Analysis of Light Response Behavior

**Figure 7:**
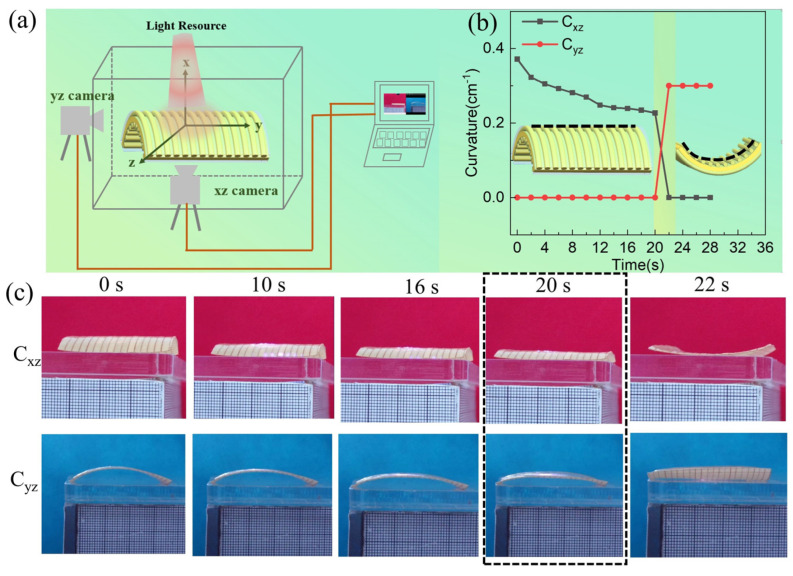
(**a**) Schematic of the experimental device established for testing the light response of the bi-stable composite antenna. (**b**) The relationship between the bending of the bi-stable composite antenna with the light-actuated time. (**c**) Photograph of the bi-stable composite antenna changing with light-actuated time.

Recently, with the introduction and implementation of energy-saving and emission-reduction measures, the application of light response actuation methods has become more and more popular. The experimental device established to analyze the light response performance of the bi-stable antenna is shown in Figure 7a. Two cameras were mounted on the xz and yz planes, respectively, to observe the curvature changes of the xz and yz planes. The reconfigurable composite antenna used to demonstrate light response behavior was 40 mm × 40 mm × 0.35 mm. The radiation layer material of the antenna used to test the light response behavior was CNTF, and the fiber linear density and fiber laying density were 100D and 5 cm^−1^, respectively. In the actuation process of the bi-stable composite antenna, the CNTF radiation layer was used as a photothermal conversion layer for the bi-stable composite antenna to convert the light response between state I and state II. The oriented-PI-fiber-reinforced PDMS layer acted as an actuating layer with photothermal sensitivity, and the electro-spun polyimide film acted as an inert layer. The wavelength of the light source for the light response experiment was 395 nm.

Figure 7b shows the relationship between the bending of the bi-stable composite antenna and the light-actuated time. It can be seen that the curvature change curves can be divided into state I stage, state II stage, and transition stage according to the state shape.

In addition, a photograph of the bi-stable composite antenna changing with light-actuated time is shown in Figure 7c. These photos show that the reconfigurable antenna exhibits significant snapping-through movements under the light. The bi-stable antenna remains in state II when the light source is removed at 22 s, and the snapping-through occurs at 20 s, indicating that the bi-stable antenna has a fast response speed and good stable-structure retention.

## 4. Conclusions

The reconfigurable antenna represents a new concept for designing antenna structures by using structurally effective materials. In this paper, a novel bi-stable composite antenna with reconfigurable performance and light-responsive behavior is realized. First, it is found that the conductivity of the antenna’s radiation layer material and the dielectric properties of the substrate layer can significantly change the antenna’s electromagnetic properties. However, the reflection coefficient curves of state I and II are coincident, indicating that the structural change has little effect on the reflection coefficient curve for antennas with the same radiation layer material. Second, the state change has a great influence on the radiation patterns; the experimental results show that the maximum radiation angles of states I and II are at 90° and 225°, respectively. The experimental gains for states I and II are 1.21 and 1.53 dB, respectively. The gain non-circularities of the antenna at states I and II are 4.48 dB and 8.35 dB, respectively. This shows that the bi-stable antenna has good omnidirectional radiation performance in state I. Finally, the bi-stable antenna is converted from state I to state II when the 395 nm ultraviolet light irradiation time is 20 s, and then the shape of state II remains unchanged. This indicates that the bi-stable antenna has fast response speed and good stable-structure retention. In summary, the work also shows that the flexible bi-stable composite structure has the characteristics of light weight, low profile, large deformation, low energy input, and multiple electromagnetic functions. It therefore has broad application prospects in deformable and multifunctional structures.

## Data Availability

Not applicable.
